# Relationships between anxiety–depression, perceived social support, and in-hospital outcomes among patients with acute myocardial infarction

**DOI:** 10.3389/fpsyt.2026.1793611

**Published:** 2026-06-16

**Authors:** Yuemei Zhu, Xiao Zhu, Yingying Zhou

**Affiliations:** Department of Cardiology, The First Affiliated Hospital of Soochow University, Suzhou, Jiangsu, China

**Keywords:** acute myocardial infarction, anxiety–depression symptoms, in-hospital outcomes, nursing evaluation, social support

## Abstract

**Objective:**

To examine associations between early anxiety–depression symptoms, perceived social support, and in-hospital outcomes in patients with acute myocardial infarction (AMI).

**Methods:**

This observational cross-sectional study enrolled 150 consecutive AMI patients. Anxiety–depression symptoms and perceived social support were assessed within 24–72 hours of admission using the Hospital Anxiety and Depression Scale (HADS) and the Perceived Social Support Scale (PSSS). Primary outcome was in-hospital complications; secondary outcomes were length of stay and sleep quality. Multivariable regression models were applied with adjustment for age, cardiac function, emergency PCI, and major comorbidities.

**Results:**

Clinically significant anxiety–depression symptoms (HADS ≥11) were present in 44.0% of patients; 27.3% developed at least one complication. Higher HADS scores were independently associated with increased complication risk, prolonged stay, and poorer sleep quality (all P < 0.05). Higher PSSS scores were associated with reduced complication risk, shorter stay, and better sleep quality (all P < 0.05). Psychosocial risk stratification demonstrated a significant gradient across all outcomes (trend P < 0.05), with results consistent across subgroup and sensitivity analyses.

**Conclusion:**

Early anxiety–depression symptoms and perceived social support are independently associated with in-hospital outcomes in AMI patients. Routine psychosocial screening may help identify high-risk patients and guide individualized nursing care.

## 1 Introduction

Acute myocardial infarction (AMI) is a severe cardiovascular condition characterized by sudden onset, rapid progression, and high risks of mortality and disability. Although reperfusion strategies, particularly emergency percutaneous coronary intervention (PCI), and evidence-based pharmacological therapy have markedly improved survival in patients with AMI, complications during hospitalization, prolonged hospital stay, and poor inpatient experience remain common. These issues adversely affect short-term recovery and healthcare resource utilization (1). Therefore, in addition to disease severity and treatment strategies, identifying other factors associated with in-hospital outcomes in patients with AMI is of clinical and nursing importance.

Beyond traditional clinical and physiological factors, psychosocial factors have received increasing attention in cardiovascular disease onset, progression, and prognosis (2). AMI is a major acute stress event and is frequently accompanied by psychological reactions. Symptoms of anxiety and depression are common among hospitalized patients (3, 4). Previous studies have shown that anxiety–depression symptom burden affects subjective well-being and quality of life. It may also impair cardiovascular stability through neuroendocrine imbalance, enhanced inflammatory responses, and behavioral changes (5, 6). However, most existing studies focus on medium- or long-term prognosis after AMI. The independent role of psychological factors in short-term in-hospital outcomes remains insufficiently evaluated (7, 8). Importantly, psychological symptoms assessed during the early stage of hospitalization may reflect the acute stress response to myocardial infarction and may directly influence treatment adherence, autonomic function, inflammatory activity, and sleep quality, thereby affecting short-term clinical outcomes (5, 9). Compared with assessments conducted during the recovery or follow-up period, early assessment provides a time-sensitive opportunity to identify high-risk patients and implement timely interventions (10). However, evidence regarding the clinical relevance of early anxiety and depression symptoms during the acute hospitalization phase remains limited.

Social support is an important psychosocial resource and is considered to have buffering and protective effects during acute illness and recovery (11). Adequate family and social support can reduce psychological stress, improve emotional status, and enhance understanding and adherence to treatment and nursing care (12). Lack of family support has also been linked to increased long-term mortality in patients with AMI (13, 14). However, studies that systematically assess social support during the acute hospitalization phase and examine its association with in-hospital outcomes are still limited (15). In addition, anxiety–depression symptoms and social support may interact with each other. Assessment from a single dimension may not fully capture psychosocial risk (16). In clinical practice, inpatient risk assessment in AMI mainly relies on disease severity and treatment response, and the role of psychosocial factors in early evaluation remains insufficiently addressed (17). Therefore, it is necessary to investigate the associations between early anxiety–depression symptom burden, social support, and in-hospital outcomes in real-world inpatient settings and to explore psychosocial risk stratification for inpatient management.

In this study, “early anxiety–depression” does not refer to previously diagnosed psychiatric disorders, but rather to the symptom burden of anxiety and depression experienced by patients during the acute phase of AMI hospitalization (within 24–72 hours after admission), as measured using the Hospital Anxiety and Depression Scale (HADS). This concept is distinct from chronic or pre-existing anxiety and depressive disorders and focuses on capturing the immediate psychological response triggered by an acute cardiovascular event.

## 2 Materials and methods

### 2.1 Study design and participants

**Figure 1:**
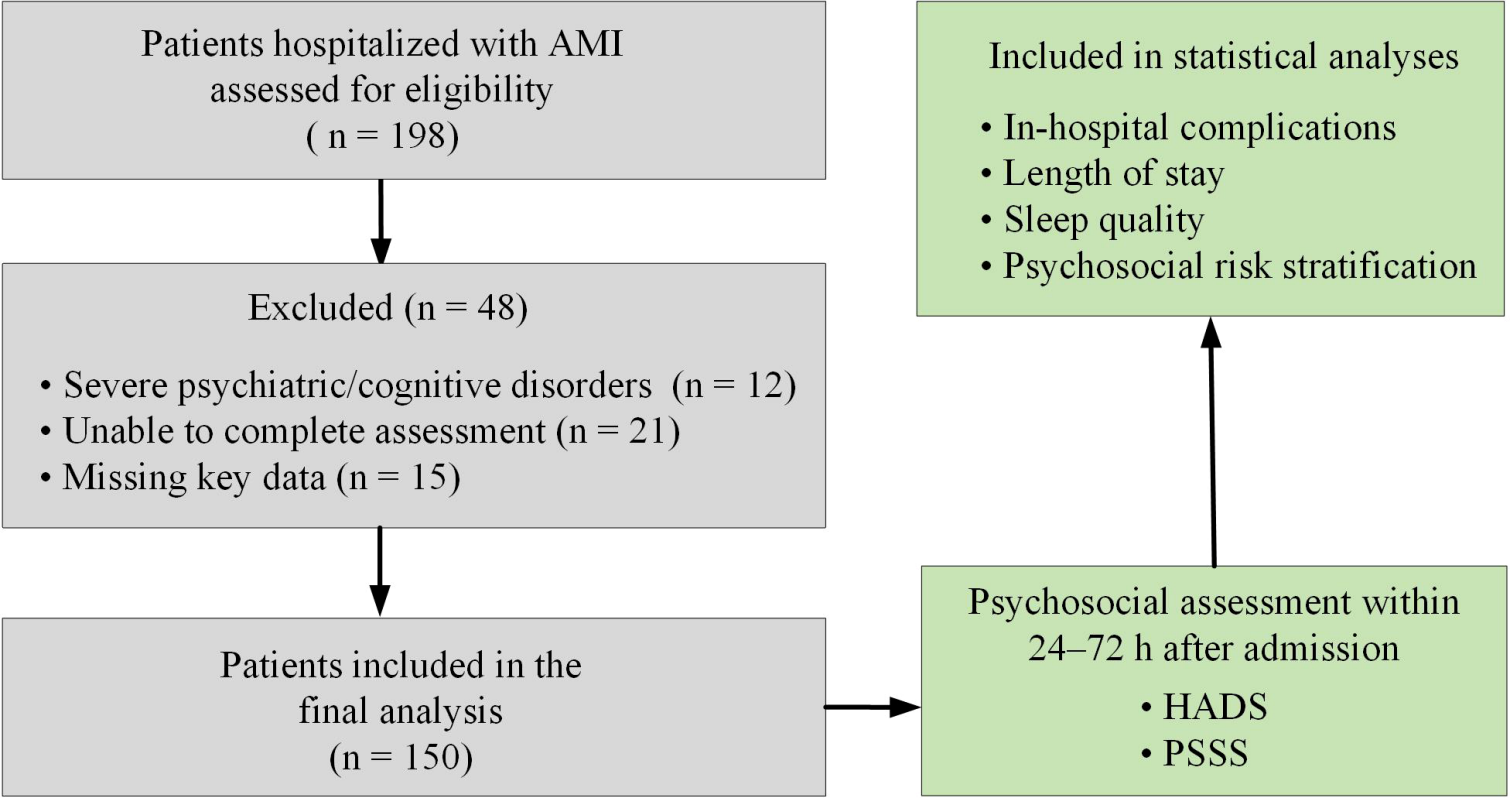
Flow diagram of patient selection and analysis.

This study employed an observational cross-sectional design. Psychosocial assessments were conducted once within 24–72 hours after admission, and in-hospital outcomes were recorded at discharge. No follow-up beyond hospitalization was performed, and all regression analyses were based on this data structure. Patients with AMI who were hospitalized in the Department of Cardiology of The First Affiliated Hospital of Soochow University between October 1, 2024 and October 1, 2025 were consecutively enrolled. A total of 150 eligible patients were included. AMI was diagnosed according to current clinical guidelines based on symptoms, electrocardiographic changes, and elevated cardiac biomarkers. Inclusion criteria were: (1) age ≥ 18 years; (2) confirmed diagnosis of AMI; (3) completion of HADS and social support assessment within 24–72 hours after admission; and (4) complete clinical and outcome data during hospitalization. Exclusion criteria were: (1) prior diagnosis of severe psychiatric disorders or cognitive impairment; (2) inability to complete psychological assessment due to critical illness or communication barriers; and (3) missing key variables or outcome data. The study protocol was approved by the Ethics Committee of The First Affiliated Hospital of Soochow University. The study used routinely documented clinical data, nursing assessment records, and psychosocial assessment data recorded as part of standard inpatient care. Access to identifiable records was restricted to authorized investigators under institutional ethical approval, and all data were de-identified prior to analysis to ensure confidentiality protection. As the study involved minimal risk to participants, no additional patient contact, and no intervention beyond routine care, the Ethics Committee waived the requirement for written informed consent. The process of patient identification, eligibility assessment, exclusion, and final inclusion is summarized in **Figure 1**.

### 2.2 Data collection

#### 2.2.1 General clinical data

Demographic and clinical data were collected, including age, sex, marital status, educational level, type of myocardial infarction (ST-segment elevation myocardial infarction [STEMI] or non-ST-segment elevation myocardial infarction [NSTEMI]), receipt of emergency PCI, and cardiac function (Killip classification, I–IV). Age was categorized into three groups (<60, 60–69, and ≥70 years) based on commonly used clinical stratification. Major comorbidities, including hypertension, diabetes mellitus, dyslipidemia, and chronic kidney disease, were recorded and identified based on documented clinical diagnoses in medical records. In addition, lifestyle-related factors, including smoking status (current smoker vs. non-current smoker), were collected. All variables were extracted from electronic medical records and nursing assessment records.

#### 2.2.2 Psychosocial measures

1. Anxiety and depression symptom assessment. Symptoms of anxiety and depression were assessed using the HADS (18). The scale contains 14 items, including the anxiety (HADS-A) and depression (HADS-D) subscales, with seven items each. Each item is scored from 0 to 3, with a total score range of 0–42. Higher scores indicate more severe symptoms. HADS scores obtained within 24–72 hours after admission were used as baseline values. A HADS total score ≥11 was considered indicative of clinically significant anxiety–depression symptoms and was used to define a high psychological symptom burden in this study (18). If multiple records were available, the earliest complete assessment was selected. In the primary analysis, the HADS total score was treated as a continuous variable to evaluate the association between each one-point increase and in-hospital outcomes.
2. Social support assessment. Perceived social support was measured using the Chinese version of the Perceived Social Support Scale (PSSS), which was originally developed by Zimet et al. and translated into Chinese by Jiang (19–21). The scale includes 12 items covering family, friend, and other support. Each item is rated from 1 to 7, yielding a total score range of 12–84. Higher scores indicate higher perceived support. PSSS scores completed within 24–72 hours after admission were used as baseline measures. The total score was entered into regression models as a continuous variable.
3. Variable categorization in sensitivity analysis. HADS scores were dichotomized as high risk (≥11) or low risk (<11) based on established scoring criteria from the original scale development (18). PSSS scores were categorized as low (≤36) or moderate-to-high (>36) social support, consistent with the standard scoring categories of the Chinese version (19–21). To reduce dependence on specific cut-offs, quartile-based stratification was also applied (22). Patients were divided into four groups (Q1–Q4) according to score distribution. The lowest-risk groups (HADS Q1; PSSS Q4) were used as reference categories.
4. Psychosocial risk stratification. A psychosocial risk stratification index was constructed based on early anxiety–depression symptoms and social support levels (2). Patients were classified into low-risk (low HADS and high PSSS), intermediate-risk (low HADS and low PSSS, or high HADS and high PSSS), and high-risk (high HADS and low PSSS) groups. This stratification was used for comparative outcome analyses.

#### 2.2.3 Outcome measures

The primary outcome was the occurrence of in-hospital complications. Complications were recorded based on predefined criteria and coded as present or absent. Events included severe arrhythmia, worsening heart failure, cardiogenic shock, and transfer to the intensive care unit.

Secondary outcomes were length of hospital stay and sleep quality. Sleep quality was assessed using Pittsburgh Sleep Quality Index (PSQI) scores routinely recorded in the nursing assessment system during hospitalization (23). Based on the sample distribution, the 33rd percentile (P33) and 67th percentile (P67) of PSQI scores were used to classify patients into good (≤P33), moderate (>P33 to ≤P67), and poor (>P67) sleep quality groups. Length of stay was defined as the number of days from admission to discharge and analyzed as a continuous variable.

### 2.3 Statistical analysis

Statistical analyses were performed using SPSS. Continuous variables were expressed as mean ± standard deviation (mean ± SD) or median (interquartile range, IQR). Categorical variables were presented as number and percentage [n (%)]. Group comparisons used the t test or Mann–Whitney U test for continuous variables and the chi-square test for categorical variables. Multivariable logistic regression was applied to in-hospital complications. Ordinal logistic regression was used for sleep quality, and multiple linear regression was used for length of stay. Potential confounders were adjusted according to study design and outcome characteristics. Basic models included age, sex, and major clinical features. Emergency PCI, Killip class, major comorbidities, and psychosocial factors were added sequentially. Trend analyses for psychosocial risk stratification were conducted using the Cochran–Armitage test or the Jonckheere–Terpstra test. Subgroup and sensitivity analyses were performed. All tests were two-sided, and P < 0.05 was considered statistically significant.

A formal *a priori* sample size calculation was not performed, as the study consecutively enrolled all eligible patients during the study period. To address this limitation, a *post-hoc* power analysis was conducted for the primary logistic regression model. The unadjusted odds ratio for clinically significant anxiety–depression symptoms (HADS ≥11 vs. <11; OR = 2.59) was derived from univariate analysis and used as the effect size estimate. With a baseline complication rate of 27.3%, two-sided α = 0.05, and 7 predictors in the final model, the minimum sample size for 80% power was estimated at approximately 75 participants. The present study enrolled 150 participants, exceeding this threshold twofold and indicating adequate statistical power. The power calculation was performed using G*Power (version 3.1.9.6). Of note, HADS and PSSS were entered as continuous variables in the primary models, yielding inherently modest per-unit odds ratios. The power estimate was therefore based on a binary categorical comparison using the prespecified cut-off, providing a more interpretable effect size reference. In multivariable regression analyses, in-hospital complications were defined as the primary outcome, with a total of 41 events observed. Given the relatively limited number of events, the number of variables included in the final models was restricted. Only clinically relevant variables that were associated with the outcome in univariate analyses were retained to reduce the risk of overfitting and ensure model stability.

## 3 Results

### 3.1 General clinical characteristics

General clinical characteristics of the 150 enrolled patients are presented in **Table 1**. Psychosocial factors and in-hospital outcomes are summarized in **Table 2**. Of note, 44.0% of patients had clinically significant anxiety–depression symptoms, 32.7% had low perceived social support, and 27.3% developed at least one in-hospital complication.

**Table 1:** Demographic and clinical characteristics of patients hospitalized with acute myocardial infarction (n = 150).

Variable	Category	n	%
Age (years)	<60	55	36.7
	60–69	52	34.7
	≥70	43	28.6
Sex	Male	112	74.7
	Female	38	25.3
Marital status	Married	128	85.3
	Unmarried/Divorced/Widowed	22	14.7
Education level	Junior high school or below	54	36.0
	High school/Technical secondary school	63	42.0
	Junior college or above	33	22.0
MI type	STEMI	96	64.0
	NSTEMI	54	36.0
Emergency PCI	Yes	104	69.3
	No	46	30.7
Killip class	I	92	61.3
	II	36	24.0
	III	15	10.0
	IV	7	4.7
Comorbidities	Hypertension	88	58.7
	Diabetes	54	36.0
	Dyslipidemia	72	48.0
	Chronic kidney disease	14	9.3
Current smoking	Yes	79	52.7
	No	71	47.3

STEMI, ST-segment elevation myocardial infarction; NSTEMI, non–ST-segment elevation myocardial infarction.

**Table 2:** Descriptive statistics of psychosocial factors and in-hospital outcomes (n = 150).

Variable	Value
HADS total score (0–42), mean ± SD	15.2 ± 7.4
HADS-A (anxiety, 0–21), mean ± SD	8.1 ± 4.1
HADS-D (depression, 0–21), mean ± SD	7.1 ± 4.3
HADS categories, n (%)	Normal (0–7): 46 (30.7%)
	Borderline (8–10): 38 (25.3%)
	Definite (≥11): 66 (44.0%)
PSSS total score (12–84), n (%)	56.8 ± 12.6
PSSS categories, n (%)	Low (≤50): 49 (32.7%)
	Moderate (51–62): 51 (34.0%)
	High (≥63): 50 (33.3%)
Any in-hospital complication, n (%)	41 (27.3%)
Complication components, n (%)	Severe arrhythmia: 18 (12.0%)
	worsening heart failure: 15 (10.0%)
	cardiogenic shock: 9 (6.0%)
	ICU transfer: 11 (7.3%)
Length of stay (days), median (IQR)	8 (6–11)
Length of stay categories, n (%)	≤7 days: 56 (37.3%)
	8–10 days: 51 (34.0%)
	≥11 days: 43 (28.7%)
Sleep quality, n (%)	Good: 59 (39.3%)
	Fair: 63 (42.0%)
	Poor: 28 (18.7%)

“Any in-hospital complication” was defined as the occurrence of at least one complication during hospitalization (n = 41). The proportions of individual complications were calculated using the total sample size (n = 150) as the denominator. As multiple complications may occur in the same patient, the sum of individual components may exceed 41.

### 3.2 Univariate analysis by in-hospital complication status

Univariate analyses indicated that age group, Killip class, chronic kidney disease, anxiety–depression level, and social support level were significantly associated with the occurrence of in-hospital complications (P < 0.05) (**Table 3**).

**Table 3:** Univariate comparisons between patients with and without in-hospital complications (n = 150).

Variable	Category	No complications (n = 109)	Complications (n = 41)	Statistic	P value
Age (years), n (%)	<60	45 (41.3)	10 (24.4)		
	60–69	39 (35.8)	13 (31.7)	χ^2^ = 8.21	0.016
	≥70	25 (22.9)	18 (43.9)		
Sex, n (%)	Male	80 (73.4)	32 (78.0)	χ^2^ = 0.33	0.565
	Female	29 (26.6)	9 (22.0)		
Marital status, n (%)	Married	95 (87.2)	33 (80.5)	χ^2^ = 1.07	0.301
	Unmarried/Divorced/Widowed	14 (12.8)	8 (19.5)		
Education level, n (%)	Junior high or below	38 (34.9)	16 (39.0)		
	High school/Technical secondary	45 (41.3)	18 (43.9)	χ^2^ = 0.86	0.651
	Junior college or above	26 (23.9)	7 (17.1)		
MI type, n (%)	STEMI	66 (60.6)	30 (73.2)	χ^2^ = 2.14	0.143
	NSTEMI	43 (39.4)	11 (26.8)		
Emergency PCI, n (%)	Yes	80 (73.4)	24 (58.5)	χ^2^ = 3.27	0.070
	No	29 (26.6)	17 (41.5)		
Killip class, n (%)	I–II	98 (89.9)	30 (73.2)	χ^2^ = 7.12	0.008
	III–IV	11 (10.1)	11 (26.8)		
Hypertension, n (%)	No	48 (44.0)	14 (34.1)		
	Yes	61 (56.0)	27 (65.9)	χ^2^ = 1.18	0.277
Diabetes, n (%)	No	73 (67.0)	23 (56.1)		
	Yes	36 (33.0)	18 (43.9)	χ^2^ = 1.61	0.205
Dyslipidemia, n (%)	No	61 (56.0)	17 (41.5)		
	Yes	48 (44.0)	24 (58.5)	χ^2^ = 2.63	0.105
Chronic kidney disease, n (%)	No	102 (93.6)	34 (82.9)		
	Yes	7 (6.4)	7 (17.1)	χ^2^ = 4.09	0.043
Current smoking, n (%)	No	53 (48.6)	18 (43.9)		
	Yes	56 (51.4)	23 (56.1)	χ^2^ = 0.26	0.609
HADS category, n (%)	<8	40 (36.7)	6 (14.6)		
	8–10	28 (25.7)	10 (24.4)	χ^2^ = 10.6	0.005
	≥11	41 (37.6)	25 (61.0)		
PSSS category, n (%)	High (≥63)	45 (41.3)	5 (12.2)		
	Moderate (51–62)	39 (35.8)	12 (29.3)	χ^2^ = 18.1	<0.001
	Low (≤50)	25 (22.9)	24 (58.5)		
Length of stay, days, median (IQR)	—	7 (6–10)	10 (8–13)	U = 1390	<0.001
Sleep quality, n (%)	Good/Fair	95 (87.2)	27 (65.9)		
	Poor	14 (12.8)	14 (34.1)	χ^2^ = 9.49	0.002

In-hospital complications were a composite outcome, defined as the occurrence of any of the following events: severe arrhythmia, worsening heart failure, cardiogenic shock, or ICU transfer. Categorical variables were compared using the chi-square test. Continuous variables were compared using the Mann–Whitney U test according to distribution. Abbreviations: STEMI, ST-segment elevation myocardial infarction; NSTEMI, non–ST-segment elevation myocardial infarction.

### 3.3 Multivariable logistic regression for in-hospital complications

Based on the univariate results, variables with P < 0.10 were included in multivariable logistic regression models. The dependent variable was the occurrence of in-hospital complications (yes/no). The analysis aimed to evaluate the independent associations between psychosocial factors and complications. Multivariable logistic regression analysis is presented in **Table 4**. After adjustment for clinical covariates, higher HADS total scores were independently associated with an increased risk of in-hospital complications, whereas higher PSSS total scores were independently associated with a reduced risk.

**Table 4:** Multivariable logistic regression analysis for in-hospital complications (n = 150).

Variable	Model 1 (OR, 95% CI)	P value	Model 2 (OR, 95% CI)	P value
Age 60–69 years	1.42 (0.63–3.18)	0.392	1.31 (0.57–3.03)	0.523
Age ≥70 years	2.56 (1.14–5.74)	0.023	2.21 (1.01–5.13)	0.046
Emergency PCI (yes)	0.62 (0.31–1.03)	0.066	0.69 (0.35–1.17)	0.128
Killip class III–IV	2.98 (1.28–6.94)	0.011	2.41 (1.02–5.72)	0.045
Chronic kidney disease	2.63 (1.01–6.83)	0.048	2.19 (0.82–5.87)	0.119
HADS total score (per 1-point increase)	—	—	1.08 (1.03–1.14)	0.002
PSSS total score (per 1-point increase)	—	—	0.96 (0.93–0.99)	0.008

Model 1 adjusted for age group, emergency PCI, Killip class, and chronic kidney disease. Model 2 further included HADS total score and PSSS total score. The same adjustment principle applied below.

### 3.4 Associations between psychosocial factors and length of hospital stay

Length of stay was treated as a continuous outcome. Multiple regression models were constructed to examine the associations between psychosocial factors and the hospitalization course. Variables with P < 0.10 in univariate analyses for length of stay were included. (**Supplementary Table 1**).

In the multivariable regression model, higher HADS total scores were independently associated with longer hospital stay, whereas higher PSSS total scores were independently associated with shorter hospitalization (both P < 0.05) (**Table 5**).

**Table 5:** Multiple regression analysis for length of stay (n = 150).

Variable	Model 1 (β, 95% CI)	P value	Model 2 (β, 95% CI)	P value
Age 60–69 years	0.72 (−0.48, 1.92)	0.238	0.61 (−0.57, 1.79)	0.312
Age ≥70 years	1.84 (0.63, 3.05)	0.003	1.52 (0.36, 2.68)	0.010
Emergency PCI (yes)	−0.96 (−2.01, 0.09)	0.073	−0.82 (−1.84, 0.20)	0.114
Killip class III–IV	2.47 (1.01, 3.93)	0.001	2.03 (0.65, 3.41)	0.004
Chronic kidney disease	2.11 (0.34, 3.88)	0.020	1.68 (−0.04, 3.40)	0.055
HADS total score (per 1-point increase)	—	—	0.18 (0.10, 0.26)	<0.001
PSSS total score (per 1-point increase)	—	—	−0.11 (−0.18, −0.04)	0.002

### 3.5 Ordinal outcome analysis for sleep quality

Univariate results showed that patients with poor sleep had significantly higher HADS total scores and significantly lower PSSS total scores (both P < 0.01). In addition, the proportion of Killip class III–IV was higher in the poor-sleep group. Length of stay was also clearly longer in this group (P < 0.05) (**Table 6**).

**Table 6:** Univariate comparisons between sleep quality groups (n = 150).

Variable	Good/Fair (n = 122)	Poor (n = 28)	Statistic	P value
Age (years), mean ± SD	62.9 ± 10.5	65.6 ± 11.4	t = 1.23	0.221
Age ≥70 years, n (%)	32 (26.2)	11 (39.3)	χ^2^ = 2.01	0.156
Emergency PCI (yes), n (%)	88 (72.1)	16 (57.1)	χ^2^ = 2.47	0.116
Killip class III–IV, n (%)	15 (12.3)	7 (25.0)	χ^2^ = 3.98	0.046
Chronic kidney disease, n (%)	9 (7.4)	5 (17.9)	χ^2^ = 3.12	0.077
HADS total score, mean ± SD	14.3 ± 6.9	19.8 ± 7.1	t = 3.86	<0.001
PSSS total score, mean ± SD	58.4 ± 11.9	49.6 ± 13.5	t = 3.39	0.001
Length of stay, days, median (IQR)	8 (6–10)	11 (9–14)	U = 1235	<0.001

After further adjustment for age group, emergency PCI, Killip class, and chronic kidney disease, ordinal logistic regression showed that higher HADS total scores remained significantly associated with poorer sleep quality. Higher PSSS total scores were significantly associated with improved sleep quality (both P < 0.05) (**Table 7**).

**Table 7:** Ordinal logistic regression results for sleep quality (n = 150).

Variable	aOR (95% CI)	P value
HADS total score (per 1-point increase)	1.09 (1.03–1.16)	0.004
PSSS total score (per 1-point increase)	0.95 (0.91–0.98)	0.002
Age ≥70 years	1.28 (0.62–2.64)	0.503
Emergency PCI (yes)	0.88 (0.45–1.74)	0.713
Killip class III–IV	1.91 (1.01–3.61)	0.047
Chronic kidney disease	1.34 (0.61–2.97)	0.468

The model adjusted for age group, emergency PCI, Killip class, and chronic kidney disease. aOR indicates adjusted odds ratio. Length of stay was treated as a process indicator and was used only for univariate comparisons; it was not entered into the ordinal logistic regression model.

### 3.6 Comparison of in-hospital outcomes across psychosocial risk strata

Participants were classified according to the psychosocial risk stratification method described in the Methods section. In-hospital outcomes were then compared across risk levels. The results showed a clear gradient pattern. As psychosocial risk increased, the rate of in-hospital complications rose. Length of stay also tended to be longer. In addition, the proportion of patients with poor sleep quality increased markedly (trend tests, P < 0.05) (**Table 8**).

**Table 8:** In-hospital outcomes across psychosocial risk strata (n = 150).

Psychosocial risk level	n	Any in-hospital complication, n (%)	Length of stay, days, median (IQR)	Poor sleep quality, n (%)
Low-risk (low HADS + high PSSS)	45	6 (13.3)	7 (6–9)	3 (6.7)
Intermediate-risk	63	16 (25.4)	8 (6–11)	11 (17.5)
High-risk (high HADS + low PSSS)	42	19 (45.2)	11 (9–14)	14 (33.3)
P for trend	—	<0.001	<0.001	0.002

### 3.7 Sensitivity analyses of associations between psychosocial factors and in-hospital outcomes

Sensitivity analyses were conducted to assess the robustness of the main findings. Because sleep quality and in-hospital complications may vary across clinical contexts, the primary analyses were repeated in predefined subgroups. After stratification by age (<70 years vs. ≥70 years) and Killip class (I–II vs. III–IV), the direction of associations remained consistent. Higher HADS total scores were still linked to higher complication risk, poorer sleep quality, and longer length of stay. Higher PSSS total scores continued to correlate with lower complication risk, shorter hospitalization, and better sleep quality. No significant interactions were observed across subgroups (interaction P > 0.05) (**Supplementary Table 2**).

In addition, when HADS and PSSS were re-modeled as categorical variables instead of continuous variables, the results were consistent with the primary analyses. Patients with higher psychosocial risk still showed worse in-hospital outcomes (**Supplementary Table 3**).

## 4 Discussion and conclusion

This observational cross-sectional study was conducted in a real-world clinical setting to examine the associations between early anxiety–depression symptom burden, social support, and in-hospital outcomes among patients hospitalized with AMI. After comprehensive adjustment for age, cardiac function class, emergency PCI, and major comorbidities, higher levels of anxiety and depressive symptoms were independently associated with a higher risk of in-hospital complications, longer length of stay, and poorer sleep quality. In contrast, higher social support was significantly related to more favorable in-hospital outcomes. In addition, a psychosocial risk stratification index based on these factors effectively differentiated in-hospital outcomes across risk levels, highlighting the relevance of psychosocial factors in AMI inpatient management.

The study showed that elevated anxiety–depression symptom burden at admission were independently linked to a higher risk of in-hospital complications, prolonged hospitalization, and worse sleep quality in patients with AMI. These associations remained stable after adjustment for age, cardiac function class, emergency PCI, and chronic kidney disease. This finding suggests that psychological symptoms are not merely accompanying features of disease severity. They may exert an independent influence on in-hospital processes and short-term outcomes. Previous studies have demonstrated the adverse impact of anxiety and depression symptoms on prognosis in patients with acute coronary syndromes (7). Several prospective investigations reported that AMI patients with depressive symptoms at admission or early during hospitalization had a higher incidence of arrhythmias, worsening heart failure, and adverse events, as well as longer hospital stays (3). Mechanistic studies suggested that symptoms of anxiety and depression may trigger autonomic imbalance, reduced heart rate variability, and enhanced inflammatory responses. These changes can lead to electrical instability and fluctuating cardiac function, thereby increasing the likelihood of complications (9). Other studies also indicated that psychological distress may impair patients’ understanding of treatment plans and cooperation with care, resulting in lower medication adherence and reduced participation in rehabilitation. This process may indirectly delay clinical stabilization (24). In the present study, the association between higher anxiety–depression symptom scores and longer hospital stay further supports the hypothesis that psychological symptoms influence short-term outcomes by affecting the in-hospital course. These findings imply that reliance on traditional clinical indicators alone may underestimate the contribution of psychological factors to in-hospital risk. It should be noted that, due to the observational cross-sectional design, the observed associations do not establish causality, and reverse causation cannot be excluded. However, the consistency of results after adjustment for disease severity indicators and across clinical subgroups suggests that psychosocial factors may reflect more than disease severity alone and may show relatively independent associations with in-hospital outcomes.

The study also demonstrated that higher social support was associated with a lower risk of in-hospital complications, shorter length of stay, and better sleep quality among AMI patients. These associations remained significant in multivariable models, indicating an independent protective effect. This observation is consistent with previous evidence linking social support to clinical outcomes in cardiovascular disease. Earlier studies showed that adequate social support can reduce psychological stress after cardiovascular events and improve prognosis by stabilizing emotional status and enhancing coping capacity (25). Research in patients with AMI and unstable angina reported that individuals with insufficient social support were more likely to experience persistent anxiety and depression symptoms and had higher risks of in-hospital complications and readmission (14). Behavioral studies further suggested that patients with stronger family and social support are more likely to follow medical advice and actively engage in rehabilitation and nursing care, which may reduce adverse in-hospital events (12). From a physiological perspective, social support may buffer stress responses, attenuate sympathetic activation, and reduce inflammatory activity, thereby improving cardiovascular stability (26). In this study, the consistent associations between PSSS scores and complications, length of stay, and sleep quality provide additional clinical evidence for the protective role of social support in AMI hospitalization. Therefore, social support represents not only a background factor for mental health but also a potential modifiable resource influencing in-hospital processes and short-term outcomes.

Beyond separate analyses of anxiety–depression symptoms and social support, a psychosocial risk stratification model was developed. The results showed a clear dose–response pattern. As psychosocial risk increased, complication rates rose, hospital stays became longer, and the proportion of patients with poor sleep quality increased. This pattern suggests that single psychological or social indicators may not fully capture overall psychosocial risk. Combined stratification appears more effective in distinguishing in-hospital outcome risk levels. Previous studies in chronic heart failure, coronary artery disease, and stroke populations applied combined psychological and social risk assessment strategies and found that integrated psychosocial scores outperformed single indicators in predicting in-hospital outcomes and functional recovery (27, 28). These studies proposed that anxiety–depression symptoms and social support do not act independently but may interact through behavioral patterns, stress responses, and adherence to care. The stratification approach used in this study was based on commonly applied clinical scales and was easy to implement. It demonstrated good discriminative ability across multiple outcomes, supporting its clinical feasibility. Building on existing evidence on psychological symptom risk assessment in acute coronary syndrome populations, this study applied a combined psychosocial stratification strategy to short-term outcomes in hospitalized AMI patients. This approach extends the application of psychosocial risk assessment to inpatient management of acute cardiovascular disease. It should be emphasized that this stratification is not intended to replace traditional clinical risk tools. Rather, it complements them. Compared with tools centered on physiological severity, such as the Killip classification, psychosocial stratification reflects potential risks related to stress coping, care participation, and inpatient experience. It may help identify high-risk patients with similar disease severity who are more likely to experience unfavorable in-hospital outcomes and thus inform stratified nursing and individualized interventions.

The findings suggest that, in AMI inpatient care, attention should extend beyond disease severity and treatment strategies. Early and systematic assessment of psychological status and social support is of clinical relevance. Incorporating anxiety–depression symptoms screening and social support evaluation into routine early nursing assessments may facilitate timely identification of psychosocially high-risk patients and guide individualized nursing and psychological interventions. Among patients with comparable disease severity and treatment strategies, such assessments may further distinguish those at higher in-hospital risk. This approach may inform comprehensive inpatient management priorities and potentially improve patient experience and short-term outcomes.

Several limitations should be acknowledged. First, this was a single-center observational cross-sectional study with a relatively small sample size, which may limit generalizability. Validation in multicenter studies with larger populations is required. Second, psychosocial factors were assessed only once during the early stage of hospitalization, which does not capture dynamic changes in psychological status and perceived social support during the hospital stay. This single time-point assessment reflects a cross-sectional analytical framework and may limit causal inference and generalizability. In addition, anxiety and depression were evaluated using the HADS, a self-reported instrument that reflects symptom levels rather than clinical diagnoses. Therefore, the results may be subject to reporting bias and may also be influenced by the acute stress response triggered by the myocardial infarction event, and thus may not fully represent patients’ long-term psychological status. Third, although multiple clinical confounders were adjusted for in the multivariable analyses, residual confounding cannot be completely excluded. Future studies using prospective designs with dynamic follow-up are needed to clarify causal relationships between psychosocial factors and short- and long-term outcomes in AMI. The findings of this study should be interpreted as exploratory.

## Data Availability

The raw data supporting the conclusions of this article will be made available by the authors, without undue reservation.
