# Supplementary material for: Relationships between anxiety–depression, perceived social support, and in-hospital outcomes among patients with acute myocardial infarction
Source: Front Psychiatry. 2026 Jun 16;17:1793611. doi: 10.3389/fpsyt.2026.1793611 (PMC13317489; doi:10.3389/fpsyt.2026.1793611)
Supplement: Supplementary file 1 [file Table1.docx]

Supplementary Material

# Supplementary Tables

**Supplementary Table 1. Univariate analyses of clinical and psychosocial factors associated with length of stay (n = 150)**

| Variable | Category | Length of stay, days, median (IQR) | Statistic | P value |
| --- | --- | --- | --- | --- |
| Age (years) | <60 | 7（6–9） |  |  |
|  | 60–69 | 8（6–11） | H = 8.94 | 0.011 |
|  | ≥70 | 10（8–13） |  |  |
| Sex | Male | 8（6–11） | U = 2416 | 0.318 |
|  | Female | 9（6–12） |  |  |
| MI type | STEMI | 9（7–12） | U = 2149 | 0.072 |
|  | NSTEMI | 8（6–10） |  |  |
| Emergency PCI | Yes | 8（6–10） | U = 2217 | 0.061 |
|  | No | 10（7–13） |  |  |
| Killip class | I–II | 7（6–10） | U = 1832 | <0.001 |
|  | III–IV | 12（9–15） |  |  |
| Chronic kidney disease | No | 8（6–11） | U = 812 | 0.018 |
|  | Yes | 11（9–14） |  |  |
| HADS total score | Continuous | r = 0.41 | — | <0.001 |
| PSSS total score | Continuous | r = −0.36 | — | <0.001 |

**Note:** Group comparisons used the Mann–Whitney U test or Kruskal–Wallis H test. Spearman correlation analysis was used for continuous variables. Abbreviations: STEMI, ST-segment elevation myocardial infarction; NSTEMI, non–ST-segment elevation myocardial infarction.

**Supplementary Table 2. Sensitivity analyses of associations between psychosocial factors and in-hospital outcomes across subgroups**

| Subgroup | Outcome | HADS total score aOR / β (95% CI) | P value | PSSS total score aOR / β (95% CI) | P value | P for interaction |
| --- | --- | --- | --- | --- | --- | --- |
| Age <70 years (n = 78) | In-hospital complications | 1.08（1.02–1.15） | 0.009 | 0.94（0.90–0.98） | 0.004 | 0.62 |
|  | Poor sleep quality | 1.11（1.04–1.19） | 0.002 | 0.93（0.89–0.97） | 0.001 | 0.58 |
|  | Length of stay (days) | β = 0.21（0.09–0.33） | 0.001 | β = −0.18（−0.29–−0.07） | 0.002 | 0.65 |
| Age ≥70 years (n = 72) | In-hospital complications | 1.10（1.03–1.18） | 0.006 | 0.95（0.91–0.99） | 0.011 |  |
|  | Poor sleep quality | 1.12（1.05–1.20） | <0.001 | 0.94（0.90–0.98） | 0.006 |  |
|  | Length of stay (days) | β = 0.24（0.11–0.37） | <0.001 | β = −0.20（−0.32–−0.09） | 0.001 |  |
| Killip I–II (n = 103) | In-hospital complications | 1.07（1.01–1.13） | 0.018 | 0.95（0.91–0.99） | 0.013 | 0.71 |
|  | Poor sleep quality | 1.09（1.03–1.16） | 0.004 | 0.94（0.90–0.98） | 0.005 | 0.69 |
|  | Length of stay (days) | β = 0.19（0.08–0.30） | 0.001 | β = −0.17（−0.28–−0.06） | 0.003 | 0.74 |
| Killip III–IV (n = 47) | In-hospital complications | 1.12（1.03–1.22） | 0.007 | 0.93（0.88–0.99） | 0.021 |  |
|  | Poor sleep quality | 1.14（1.05–1.24） | 0.002 | 0.92（0.87–0.98） | 0.009 |  |
|  | Length of stay (days) | β = 0.27（0.12–0.42） | <0.001 | β = −0.22（−0.38–−0.07） | 0.005 |  |

**Note:** All models were adjusted according to the multivariable strategy described in the Methods. Depending on the outcome, the models adjusted for age, sex, Killip class, emergency PCI, comorbidities, and other potential confounders. aOR indicates adjusted odds ratio. The interaction P value assessed statistical heterogeneity of the psychosocial associations across subgroups.

**Supplementary Table 3. Sensitivity analyses under different variable specifications**

| Variable specification | Outcome | High HADS group aOR / β (95% CI) | P value | Low PSSS group aOR / β (95% CI) | P value |
| --- | --- | --- | --- | --- | --- |
| Continuous variables (primary analysis) | Any in-hospital complication | 1.09（1.03–1.16） | 0.004 | 0.95（0.91–0.98） | 0.002 |
|  | Poor sleep quality | 1.11（1.05–1.18） | <0.001 | 0.94（0.90–0.98） | 0.004 |
|  | Length of stay (days) | β = 0.23（0.12–0.34） | <0.001 | β = −0.19（−0.31–−0.07） | 0.002 |
| Categorical variables (quartiles) | Any in-hospital complication | 2.36（1.28–4.35） | 0.006 | 0.48（0.26–0.89） | 0.020 |
|  | Poor sleep quality | 2.91（1.54–5.51） | 0.001 | 0.44（0.23–0.85） | 0.014 |
|  | Length of stay (days) | β = 2.10（0.98–3.22） | <0.001 | β = −1.84（−3.01–−0.67） | 0.002 |

**Note:** Covariates and the adjustment strategy were the same as in the primary analysis. Continuous models included the total scale scores. Categorical models used quartile-based grouping.
